# Supplementary material for: Onvansertib-Based Second-Line Therapies in Combination with Gemcitabine and Carboplatin in Patient-Derived Platinum-Resistant Ovarian Carcinomas
Source: Int J Mol Sci. 2025 Jun 14;26(12):5708. doi: 10.3390/ijms26125708 (PMC12193480; doi:10.3390/ijms26125708)
Supplement: Supplementary file 1 [file ijms-26-05708-s001.zip › ijms-3568111-supplementary.pdf]

Onvansertib-Based Second Line Therapies in Combination with Gemcitabine and Carboplatin in Patient Derived-Platinum Resistant Ovarian Carcinomas

Federica Guffanti[1], Ilaria Mengoli[1], Francesca Ricci[1], Ludovica Perotti[1], Elena Capellini[1], Laura Sala[2, 3], Simone Canesi[2, 3], Chu-Chiao Wu[4], Robert Fruscio[5,6], Maya Ridinger[4], Giovanna Damia[1*], Michela Chiappa[1].

1 Laboratory of Gynecological Preclinical Oncology, Experimental Oncology Department, Istituto di Ri-cerche Farmacologiche Mario Negri IRCCS, Milan, Italy; federica.guffanti@marionegri.it (F.G.); ilaria.mengoli@marionegri.it (I.M.); francesca.ricci@marionegri.it (F.R.); ludovica.perotti@marionegri.it (L.P.); elena.capellini@marionegri.it (E.C.); michela.chiappa@marionegri.it (M.C.)
2 Mouse & Animal Pathology Laboratory (MAPLab), Fondazione UniMi, Milano, Italy; laura.sala2@unimi.it (L.S.); simone.canesi@unimi.it (S.C.)
3 Department of Veterinary Medicine and Animal Sciences, University of Milano, Lodi, Italy
4 R&D Department, Cardiff Oncology, San Diego, CA, USA; cwu@cardiffoncology.com (C.-C.W.); mridinger@cardiffoncology.com (M.R.)
5 UO Gynecology, Fondazione IRCCS San Gerardo dei Tintori, Monza Italy; robert.fruscio@unimib.it
6 Department of Medicine and Surgery, University of Milan-Bicocca, Milan, Italy
* Correspondence: giovanna.damia@marionegri.it

Western Blott Analysis. Snap-frozen tumor fragments were lysed in ice-cold cell extract buffer containing 50 mM TrisHCl pH 7.4, 250 mM NaCl, 0.1% Nonidet NP40, 5 mM EDTA and NaF 50 mM with a protease inhibitor cocktail (Sigma). Protein (40 µg) were resolved on 12% SDS-PAGE gels, transferred to nitrocellulose membranes (PROTRAN, Schleicher and Shull) and immunoblotting carried out with the following antibodies and visualized using Odyssey FC Imaging System (Li-COR): anti-βactin (sc-47778, Santa Cruz Biotechnology); anti-phospho-Histone H3 (Ser10) (6G3) (#9706, Cell Signaling Technology); anti- phospho-H2AX (pSer139) (#9718, Cell Signaling); anti-rabbit and anti-mouse (#1706515, #1706516, Bio-Rad Laboratories S.r.l.) secondary antibodies.

Immunohistochemistry. 4 um thick sections were obtained at the microtome and immunolabelled. Specifically, sections underwent deparaffinization and heat-induced epitope retrieval in a water bath for 40 minutes at 100°C (Dewax and HIER Buffer H, Thermo Scientific Lab Vision, cat. No. TA-999-DHBH). Slides were rinsed in phosphate-buffered saline 1X and placed in an autostainer (Lab Vision Autostainer 480S-2D Thermo Fisher Scientific) after application of PapPen (Liquid Daido Sangyo Co. Ltd.). Endogenous peroxidase activity was blocked by incubating sections with 3% hydrogen peroxide for 10 minutes. Slides were rinsed, incubated with phosphate-buffered saline containing 10% normal goat serum for 30 minutes at room temperature to prevent nonspecific background and then incubated for 1.30 hour at room temperature with primary antibodies (Ki67 sp6 (Thermoscientific, #RM-9106-S, 1:500) and Cleaved Caspase-3 (Cell Signaling, Asp175, #9661, 1:2000). Sections were subsequently rinsed with phosphate-buffered saline 1X and incubated with a biotinylated goat anti-rabbit secondary antibody (Vector Laboratories, USA, cat. No. BA-1000) for 30 minutes and labeled through avidin-biotin-peroxidase procedure for 30 minutes (VECTASTAIN Elite ABC-Peroxidase Kit Standard, Vector Laboratories, USA, cat. No.PK-6100). The immunoreaction was visualized with 3,3′-diaminobenzidine substrate after 5 minutes incubation (DAB, Peroxidase DAB Substrate Kit, Vector Laboratories, USA, cat. No. SK-4105). Sections were counterstained with Mayer's hematoxylin for 1 minute, dehydrated in a graded alcohol series, cleared in xylene, and coverslipped with resinous mounting medium. Adequate positive controls were included in each immunolabeling assay.
Hhistological sections stained with H&E were examined with a light microscope for the detection of lesions and for the quantification of: 1) the necrosis (expressed as percentage of the necrotic area(s) in relation to the total area of the section); 2) the apoptotic index (based on the number of apoptotic figures detected in three randomly selected high power microscopic fields). Blinded evaluation of histological slides was carried out (i.e., without information about the experimental groups)

Digital Image Analysis: Immunostained slides were checked for good quality and subsequently digitized through the NanoZoomer S60 Digital slide scanner (Hamamatsu Photonics K.K., Hamamatsu City, Japan) at UNITECH NoLimits (Università degli Studi di Milano, Italy). Optimal and homogeneous parameters of acquisition were applied to scan all the samples of the study. Digitized slides were visualized using the NDP.view2 Viewing software (Hamamatsu, U12388-01). Regions of interest were collected by manually annotating vital tumor (i.e. discarding necrosis and inflammatory infiltrates if present) using the QuPath v0.5.1 software. Digital Image Analysis was performed to automatically quantify the number of Ki67/Cleaved Caspase-3 positive cells through the 'Positive cell detection' tool. Parameters used to quantify Cleaved Caspase-3 marker for samples belonging to PDX #266R and #315 are reported in the table below.

Setup parameters	
Detection image	Hematoxylin OD
Requested pixel size	0.5 um
Nucleus parameters	
Background radius	8 um
Median filter radius	2 um
Sigma	1.5 um
Minimum area	10 um^2
Maximum area	400 um^2
Intensity parameters	
Threshold	0.05
Max background intensity	2
Cell parameters	
Cell expansion	5 um
Include nucleus	Yes
General parameters	
Smooth boundaries	Yes
Make measurements	Yes
Intensity threshold parameters	
Score compartment	Nucleus: DAB OD mean
Threshold	0.2

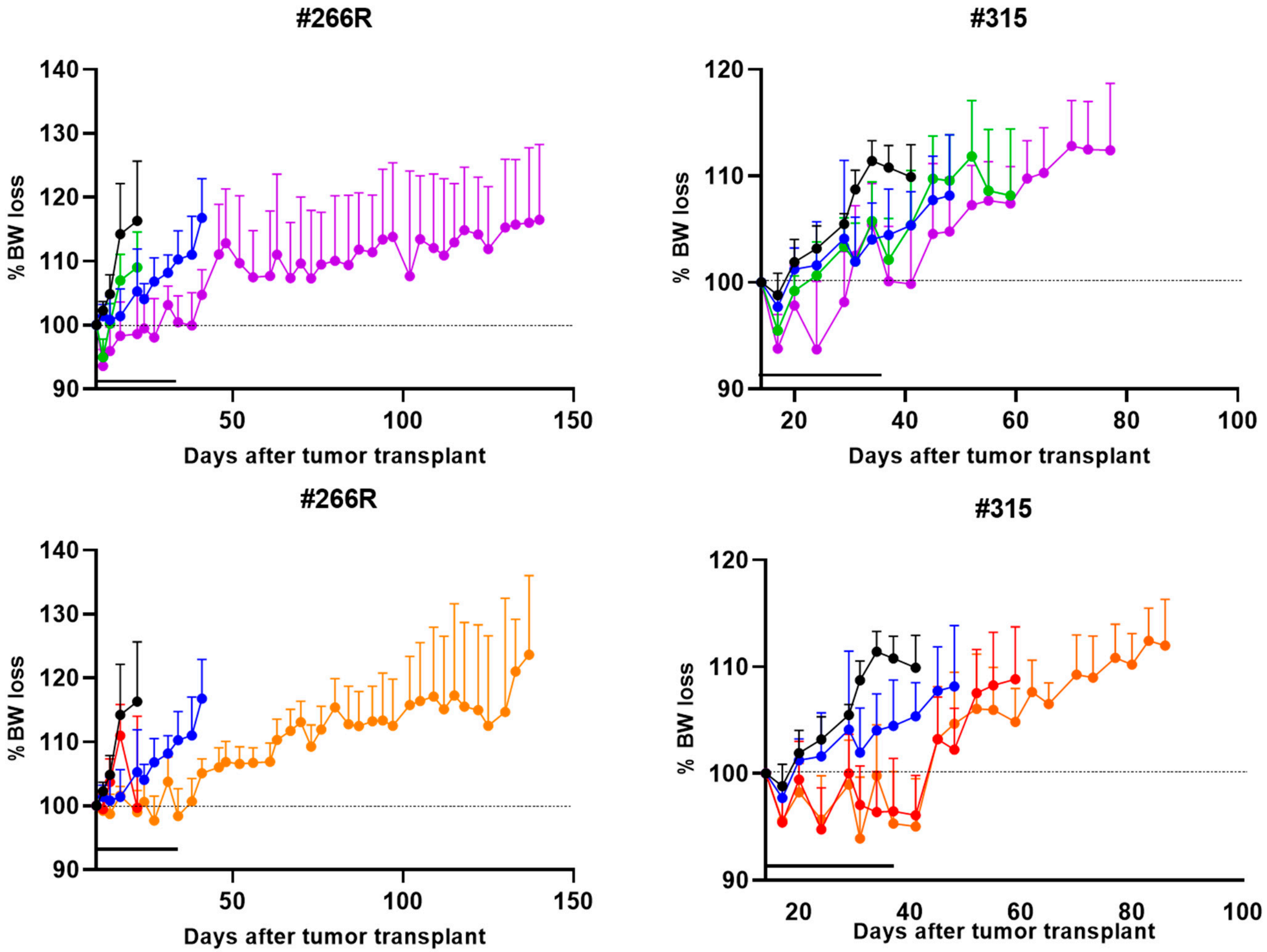

Supplementary Figure S1. Body weights from #266R and #315 tumor-bearing mice. Mice transplanted with #266R (left panel) and #315 (right panel) were treated as described in material and methods with vehicle (CTR, black curves), onvansertib 40mg/kg (ONV- blue), gemcitabine 60mg/kg (GEM- green), carboplatin 50mg/kg (CARBO- red), onvansertib+gemcitabine (ONV+GEM- violet) or onvansertib+carboplatin (ONV+CARBO- orange). Mice body weights were recorded every three days. Data represent the mean ± SD of 8-10 mice per group.

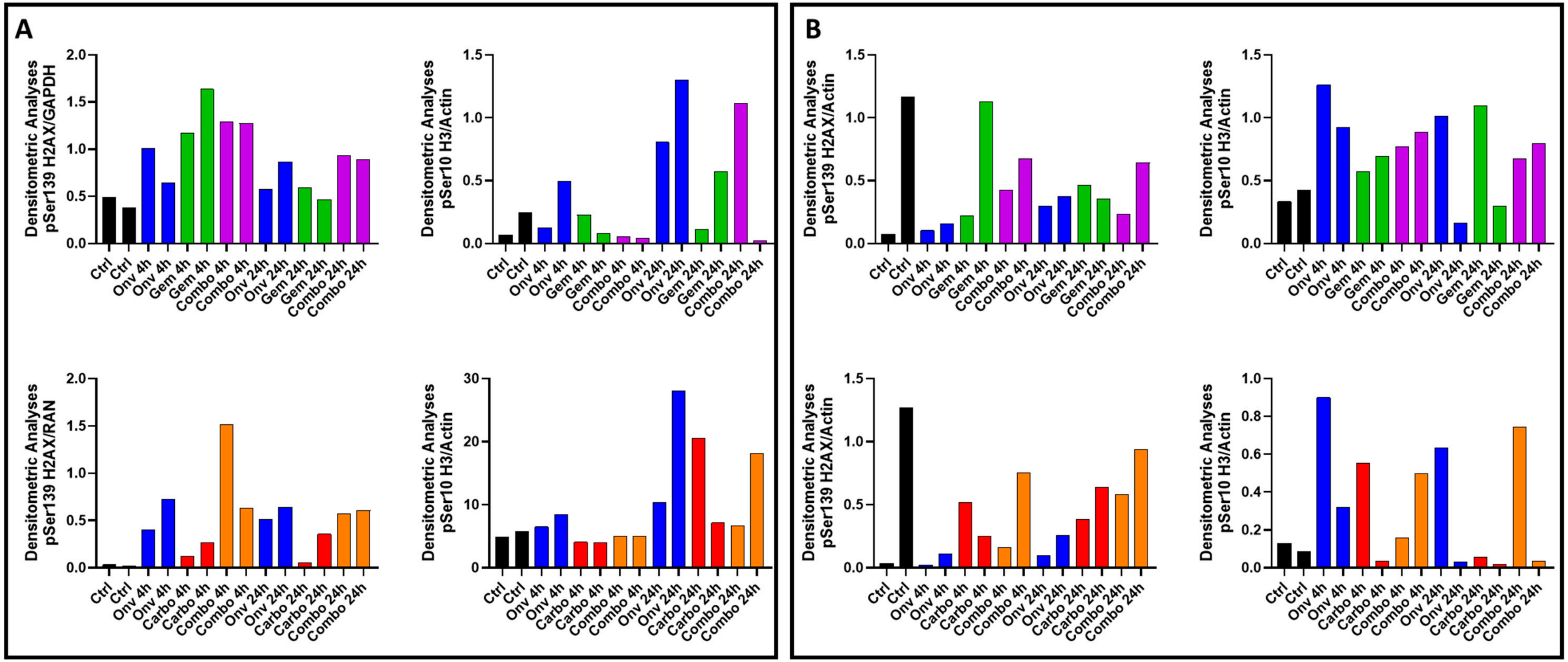

Supplementary Figure S2. Western blots densitometric analyses. Quantification of pSer10 H3 (pH3) and pSer139 H2AX (γH2AX) protein levels by densitometric analyses in #266R (panel A) and #315 (panel B) tumors treated with onvansertib/gemcitabine combination (upper panel) or onvansertib/carboplatin combination (lower panel). Protein expression was normalized over the corresponding housekeeping gene reported on y-axe.

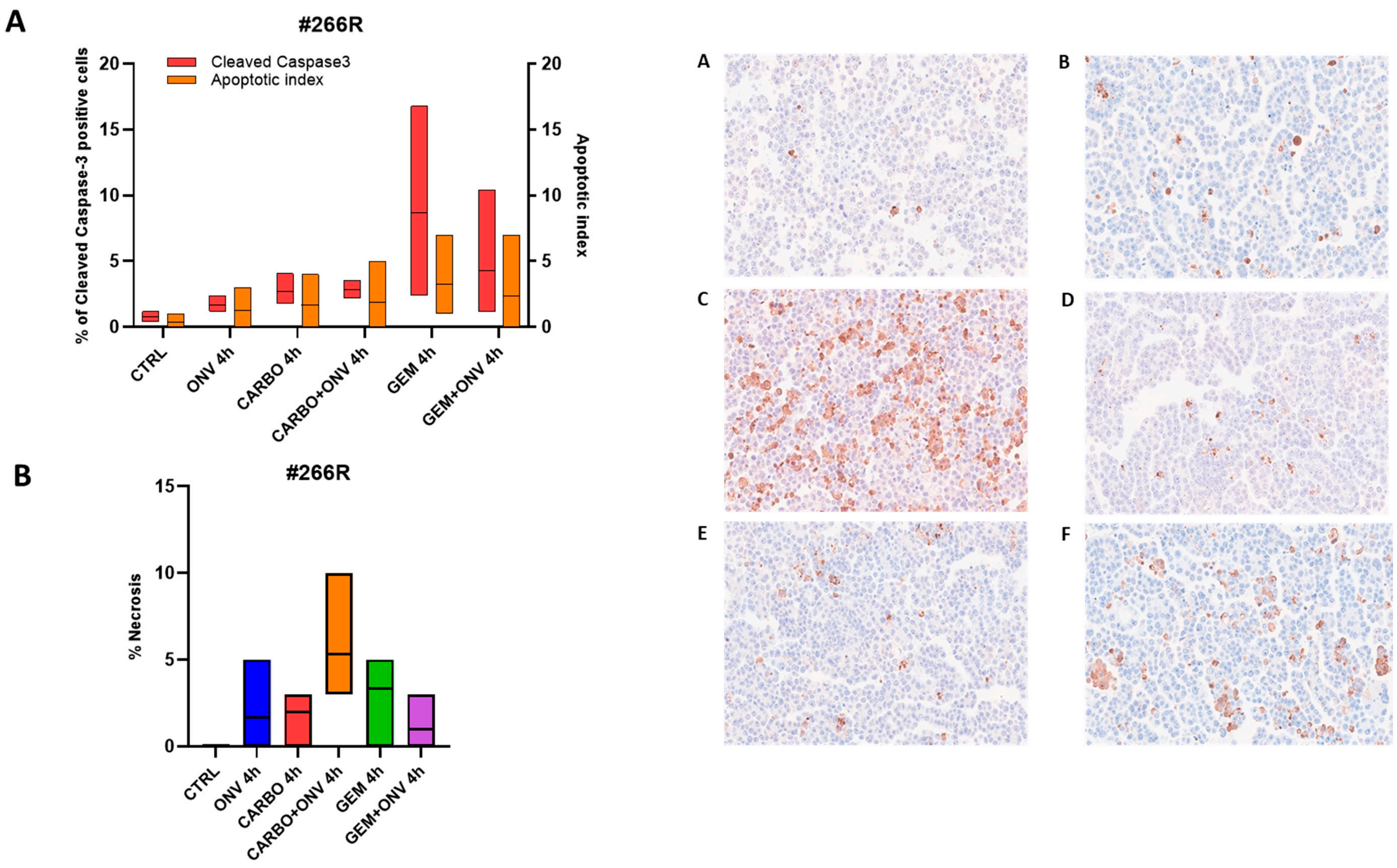

Supplementary Figure S3. IHC analyses of Apoptosis, Cleaved Caspase-3 and Necrosis in the #266R model. **A**. Quantification of the percentage of Cleaved caspase-3 positive cells and apoptotic index in FFPE tumor samples of the different experimental groups. Representative images of cleaved caspase-3 immunolabelling in CTRL (A), CARBO 4h (B), GEM 4h (C), ONV 4h (D), GEM+ONV 4h (E), and CARBO+ONV 4h (F) tumors are reported (magnitude 40x). **B**. Quantification of the percentage of necrotic cells in FFPE tumor samples of the different experimental groups.

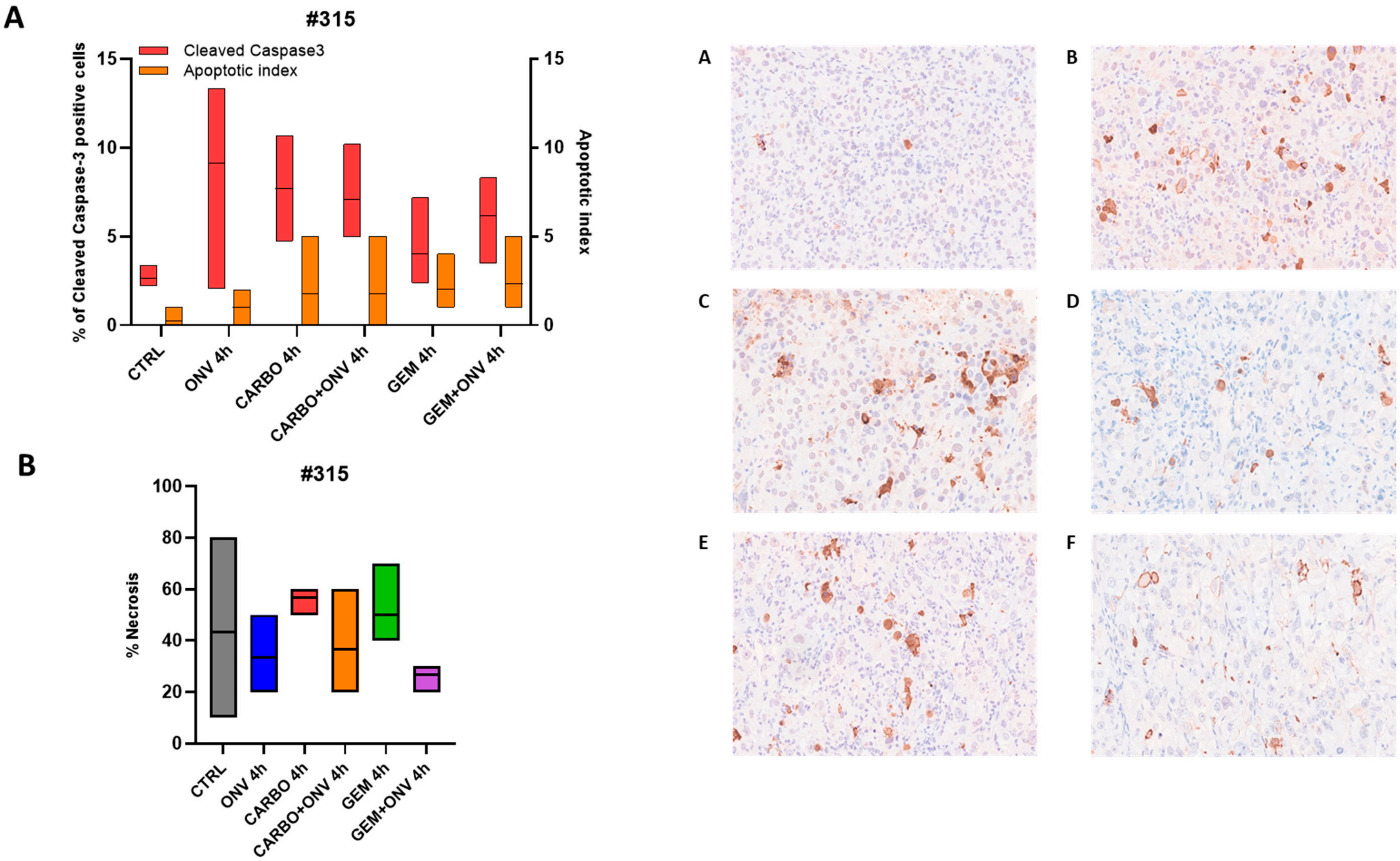

Supplementary Figure S4. IHC analyses of Apoptosis, Cleaved Caspase-3 and Necrosis in the #315 model. A. Quantification of the percentage of Cleaved caspase-3 positive cells and apoptotic index in FFPE tumor samples of the different experimental groups. Representative images of cleaved caspase-3 immunolabelling in CTRL (A), CARBO 4h (B), GEM 4h (C), ONV 4h (D), GEM+ONV 4h (E), and CARBO+ONV 4h (F) tumors are reported (magnitude 40x). **B.** Quantification of the percentage of necrotic cells in FFPE tumor samples of the different experimental groups.
